# Structure of the Assemblages of Spiders in Mediterranean Pear Orchards and the Effect of Intensity of Spraying

**DOI:** 10.3390/insects11090553

**Published:** 2020-08-20

**Authors:** Luis de Pedro, María Carmen Ortín-Angulo, Jesús Miñano, Elena López-Gallego, Juan Antonio Sanchez

**Affiliations:** 1 Department of Crop Protection, Biological Control and Ecosystem Services Laboratory, Instituto Murciano de Investigación y Desarrollo Agrario y Alimentario, C/ Mayor s/n, La Alberca, 30150 Murcia, Spain; luis.depedro@carm.es (L.d.P.); mcortinangulo@gmail.com (M.C.O.-A.); elena.lopez5@carm.es (E.L.-G.); 2 Department of Ecology and Hidrology, Campus de Espinardo, Universidad de Murcia, 30100 Murcia, Spain; jmm@um.es

**Keywords:** spiders, pear pests, pesticides, natural enemies, biological control, chemical control, population dynamics, spider guilds, wandering spiders, web builders

## Abstract

**Simple Summary:**

Spiders are one of the most abundant predatory arthropods in fruit tree orchards, where they contribute to pest control. The application of pesticides in these orchards has been largely reported to negatively affect spiders, lowering their abundance and diversity. In this study, we described the structure of the community of spiders in four Mediterranean pear orchards, and we assessed the effect of the intensity of spraying on the spider assemblages. Two of the orchards had low-intensity spraying of pesticides and the other two were sprayed more intensively. Spiders were generally more abundant and diverse in the orchards with low intensity of spraying. Moreover, the impact of the intensity of spraying varied depending on the family of spiders, with only some families of wandering spiders being significantly affected by the intensity of spraying. This suggests that the impact of pesticides on spider could be explained, at least in part, by their foraging mode. However, other local environmental factors apart from the intensity of spraying may have accounted for the differences in abundance and structure of the spider assemblages registered in pear orchards in the present research.

**Abstract:**

Spiders are key predatory arthropods that are negatively affected by spraying pesticides in orchards. The aim of this research was to determine the structure of the community of spiders in pear orchards and the impact of the intensity of spraying. The study was carried out over three years in four pear orchards in southern Spain; two of them were conducted by ourselves with no or low-intensity spraying of insecticides, and two under the criteria of technicians (conventional). Spiders were sampled on pear trees by the beating method. The orchards hosted a rich community of spiders belonging to 13 different families and 51 genera. However, the genera *Philodromus*, *Oxyopes*, *Cheiracanthium*, *Icius,* and *Neoscona* accounted for 72% of the captures. Spiders were more abundant and had a higher richness of genera in the low-intensity spraying than in conventional orchards. Philodromidae, Salticidae, and Cheiracanthiidae experienced a significant population reduction in conventional orchards, while Araneidae, Linyphiidae, and Thomisidae were not significantly affected by the intensity of spraying. The wandering hunting mode could explain the negative impact on Philodromidae, Salticidae, and Cheiracanthiidae but does not explain the lack of effect on Oxyopidae and Thomisidae. No significant effect was found on any family of web builders.

## 1. Introduction

Spiders (order Araneae) are one of the most numerous groups of arthropods in terrestrial ecosystems, with more than 45,000 described species [1,2]. In agroecosystems and fruit tree orchards, spiders are one of the most abundant and diversified groups of predators [3,4,5]. Traditionally, given their lack of specific response to a specific pest species, spiders have received less attention than specialist natural enemies, which better fit the classical role of effective natural enemies in biological control [6,7]. However, they are “well reputed” predators of many pest species of economic importance [8,9,10,11,12,13]. Spider assemblages are structured in different guilds and can exert strong predation pressure on insect pest populations [14], thus constituting an essential component of the complex of predators in integrated pest management (IPM) systems.

Around 2% of agricultural land in the European Union (EU) is occupied by orchards, with more than 3.4 million ha dedicated to fruit growing. In the EU, pear (*Pyrus communis* L. (Rosaceae)) is one of the most important fruit crops, with more than 116,000 ha devoted to pear production in 2018 [15]. Most of this land is in the Mediterranean Area, with Italy, Spain, and Portugal together having more than half of the area dedicated to pear orchards in the EU [15,16]. The great relevance of pear orchards in these regions has led to a growing awareness of the need to develop efficient pest control methods. Currently, the pear psyllid *Cacopsylla pyri* (L.) (Hemiptera: Psyllidae) is the main pest of European pear orchards [17,18,19,20,21]. Traditionally, pest control in pear orchards has relied on insecticides but due to increased restrictions concerning the application of chemicals and the development of resistance, integrated pest management (IPM) has become the most successful alternative [20,22,23]. This strategy incorporates natural enemies into decision-making and the use of compatible tactics that preserve these agents [24]. In recent years, several studies aiming to understand the role of native natural enemies in regulation of the pear psyllid population in the Mediterranean area have been carried out [20,21,25,26]. However, the knowledge of the assemblages of spiders in Mediterranean pear orchards is still extremely poor. The knowledge of the faunistics of this group of predators is extensive for the temperate European apple and pear orchards [4,27,28,29], but it is lacking for the Mediterranean area [3,30,31].

The disturbance caused by using pesticides is one of the most important and studied factors affecting spiders in agroecosystems. Many studies in European pome fruit orchards [4,7,30,32,33,34], together with studies in other areas [35,36], have assessed the effect of pesticide applications on spider communities in orchards. These studies have provided strong evidence that pesticides, particularly broad-spectrum formulations, have detrimental effects on spiders, lowering species richness and their abundance [37,38,39]. This damaging effect has even been observed for some moderately toxic pesticides that are widely employed in IPM [29,40]. The pome orchards’ arachnofauna is composed of different spider guilds that differ in their susceptibility to pesticides due to a number of behavioral factors, including the foraging mode [33,35]. Circadian activity rhythms and hunting strategies are some of the components of behavior that determine the susceptibility of spiders to pesticides. For example, Pekár [32] reported that in chemically treated orchards, some web-building species were more numerous than hunting species, since the former seem to be protected from direct spray by their web. Conversely, nocturnal species are usually exposed only to residues, while diurnal species also receive the physical impact of spraying. This suggests that pesticides may have an impact on both the abundance of populations and the structure of the community of spiders in orchards. Indeed, Bogya et al. [4] stated that pesticide application, together with other related factors, such as prey density and migration from the herbaceous layer and surroundings of the orchard, were among the main factors affecting the composition of spider communities in the canopies of fruit orchards.

Against this background, the aim of the present study was to determine the composition of the spider assemblage associated with the canopy of Mediterranean pear orchards, as well as to assess the impact of the intensity of spraying on the abundance and structure of the spider community. It was hypothesized that the use of insecticides would have a significant impact on spiders, affecting the composition of assemblages and reducing abundance and richness.

## 2. Materials and Methods

### 2.1. Location, Setting, and Management of the Orchards

The present study was carried out between 2008 and 2010 in four commercial pear orchards of approximately one hectare each, situated in two different localities in the municipality of Jumilla, Murcia, Spain. Localities were separated by approximately four kilometers, with the two orchards of the same locality approximately one kilometer apart. Each orchard had 10 lines of 200 trees trained as espaliers, with a 4 m separation between lines and 0.8 m between trees within lines. Orchards in the first locality (Loc1) and the second locality (Loc2) were planted with pear trees, *Pyrus communis* L. (cv. Ercolini), in 2001 and 2004, respectively. One line of the cultivar ‘Castell’ was planted every four lines of ‘Ercolini’ for cross-pollination.

Pest control in one of the orchards from each locality was managed by ourselves with no or limited use of insecticides. These orchards were sprayed as little as possible without compromising yield, as the study was carried out in commercial orchards, and when it was necessary, products with low persistence, such as paraffin oil, were used (Supplementary Material, Table S1). Before 2006, these orchards were managed under conventional chemical pest control, while in 2007, no pesticides were sprayed at all. The second orchard of each locality was conducted according to the criteria of growers-technicians. Before the beginning of the experiment, pest control in these orchards was effectuated roughly in the same way as in 2008. For convenience, the orchards with restricted spraying will be called “low-intensity spraying orchards—LISO” and those managed by growers/technicians will be referred to as “conventional—CO”. The spraying calendar of each orchard is given as Supplementary Material (Table S1). In all orchards, mating disruption was used to control the codling moth (*Cydia pomonella* (L.) (Lepidoptera: Tortricidae)).

### 2.2. Sampling of Spiders

Sampling of spiders on the canopy of pear trees was carried out by the beating of branches on entomological funnels [21] from March 2008 to November 2010. The orchards were sampled weekly from the beginning of March until the end of August and fortnightly during the rest of the year. Branches 2–4 cm in diameter were selected from individual trees chosen at random and hit three times at their base with a wooden stick over a funnel (28-cm-diameter) with a 100-mL plastic bottle at the bottom to collect the sample. The number of branches sampled per plot and date was 60 in 2008, and 90 in 2009 and 2010. The samples were taken to the laboratory in refrigerated containers to avoid deterioration, preserved in 70% ethanol, and observed under a stereomicroscope for counting and identification of spiders. The specimens were identified to the genus level by specialists following the criteria of Platnick et al. [2]. The reference collection of voucher specimens is held by the IMIDA (Instituto Murciano de Investigación y Desarrollo Agrario y Alimentario).

### 2.3. Data Analyses

#### 2.3.1. Analysis of the Richness of Genera

The effect of the intensity of spraying (i.e., LISO/CO) and year on the number of spider genera (richness) collected during the annual period was tested by a two-way ANOVA using the function “aov”, and separation of the means was performed with “TukeyHSD”, both functions were available in the “stats” package [41] in R (R Foundation for Statistical Computing, Vienna, Austria). The normality of the data was assessed by representing the empirical quantiles of the dependent variable against the theoretical quantiles of the normal distribution using the “qqp” function in the “car” package [41].

#### 2.3.2. Analysis of the Abundance and Population Dynamics of Spiders

Generalized linear mixed models (GLMM) were used to test for the overall effect of the intensity of spraying and year on the abundance of spiders throughout the sampling period. Because of the low captures of spiders, on each sampling event (i.e., date and orchard), the abundance of spiders was given as the number of individuals from the beating of 90 branches. As the sample size in 2008 was 60 branches, the captures in this year were standardized to 90 branches by dividing the number of spiders by 60 and multiplying it by 90. GLMM were run using the function “glmmPQL” (“MASS” package) [42] set to the quasipoisson distribution to account for over-dispersion of the data [41]. The intensity of spraying and year were introduced in the models as fixed factors and locality and date as random factors. χ^2−^ and *p*-values for the fixed factors were obtained by the Wald test using the “Anova” function in the R “car” package [41]. The post-hoc pairwise multiple comparison between the treatments (i.e., intensity of spraying*year) was run using Tukey’s test with the function “glht” in the “multcomp” package [43]. The predictions of the models were validated against the experimental values by the Pearson’s correlation test using the function “cor.test” (package “stats”) in R [41].

GLMM, following the procedure explained in the previous paragraph, were also used to test which families or guilds of spiders accounted for the putative differences in the abundance of spiders between low-intensity spraying and conventional orchards. The monthly average of the number of individuals in the different spider families or guilds in the beating of 90 branches were introduced in the models as fixed factors, and locality and month as random factors. Guilds of spiders were established following the criteria of Uetz et al. [44] (Table 1).

#### 2.3.3. Analysis of the Structure of Spider Assemblages

Redundancy analyses (RDA) were applied to find out how much of the variance in the experimental data was explained by the intensity of spraying and locality, as well as the association of different spider families with these two variables. The monthly average of the number of individuals in the different spider families in the beating of 90 branches were introduced in the models as dependent variables. Year and month were introduced in the models as covariates. The RDA analyses were performed with the function “rda” in the “vegan” package in R [41]. The significance of the terms and axes were tested with the permutation test running the function “anova.cca” (“vegan” package) for 999 permutations [41]. For all statistical analyses, the significance level was established at α = 0.05.

## 3. Results

### 3.1. Samples, Composition, and Richness of the Spider Assemblage

In the present study, 2352 spiders were collected, with most of these specimens being juveniles (91.5%). Among them, 2234 specimens were identified to the genus level, belonging to 13 families and 51 different genera. Overall, the most abundant families were Philodromidae (29.2%), Oxyopidae (18.1%), Salticidae (13.9%), Cheiracanthiidae (11.0%), and Theridiidae (9.6%) (Table 1).

*Philodromus* Walcknaer was the most abundant genus (27.8%), followed by *Oxyopes* Latreille (18.1%), *Cheiracanthium* C.L. Koch (11.0%), *Icius* Simon (8.5%), and *Neoscona* Simon (6.4%) (Supplementary Material, Table S2). Together, these genera represented 71.8% of the total number of specimens identified to the genus level. Regarding their habits, wandering spiders represented most of the captures (79.1%), with web builders constituting only 20.9% of the specimens. The classification of spiders into guilds, following the criteria of Uetz et al. [44], revealed that ambushers (35.4%) and stalkers (32.0%) were the most abundant groups of wandering spiders, and space web builders were the most abundant among web-building spiders, representing 9.6% of the specimens identified (Table 1).

**Figure 1:**
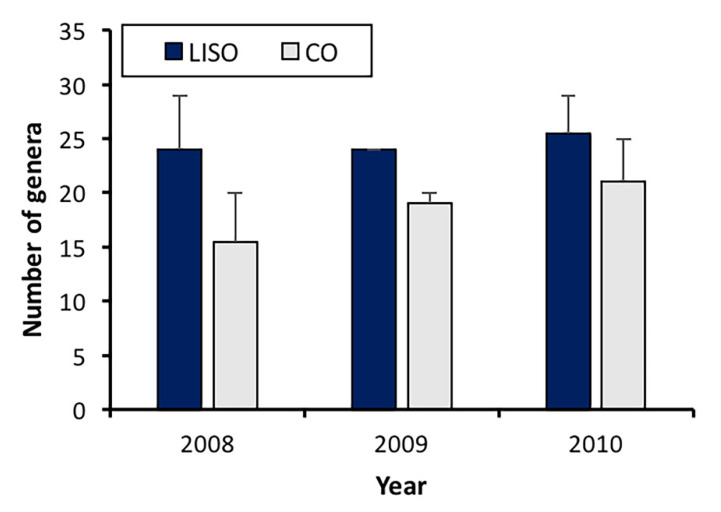
Annual richness of spider genera (mean ± SE) in pear orchards. LISO, low-intensity spraying orchards; CO, conventional orchards.

The richness of spider genera was significantly higher in the low-intensity spraying orchards (LISO) than in conventional orchards (F = 5.45, df = 1, 8, *p* = 0.048), but no significant differences were found among years (F = 0.618, df = 2, 8, *p* = 0.563). The number of spider genera collected in LISO was similar throughout the three years of the study period, with the highest value being registered in 2010 (25.5 ± 3.5, mean ± SE) (Figure 1). In conventional orchards, the number of genera increased slightly from 2008 (15.5 ± 4.5) to 2010 (21.0 ± 4.0) (Figure 1).

### 3.2. Population Dynamics of Spiders

The overall abundance of spiders was significantly higher in LISO than in conventional orchards (χ^2^ = 101.9, df = 1, *p* < 0.001), without significant differences among years (χ^2^ = 2.15, df = 2, *p* = 0.537). A significant interaction between year and intensity of spraying was found (χ^2^ = 54.54, df = 2, *p* < 0.001). In 2008, the abundance of spiders did not differ significantly between LISO and conventional orchards (Tukey’s test, *p* = 0.975), while significant differences were found in 2009 and 2010 (*p* < 0.001). A high degree of correlation was found between the prediction of the GLMM and the experimental data (coefficient = 0.895, *t*-value = 40.43, df = 406, *p* < 0.001).

**Figure 2:**
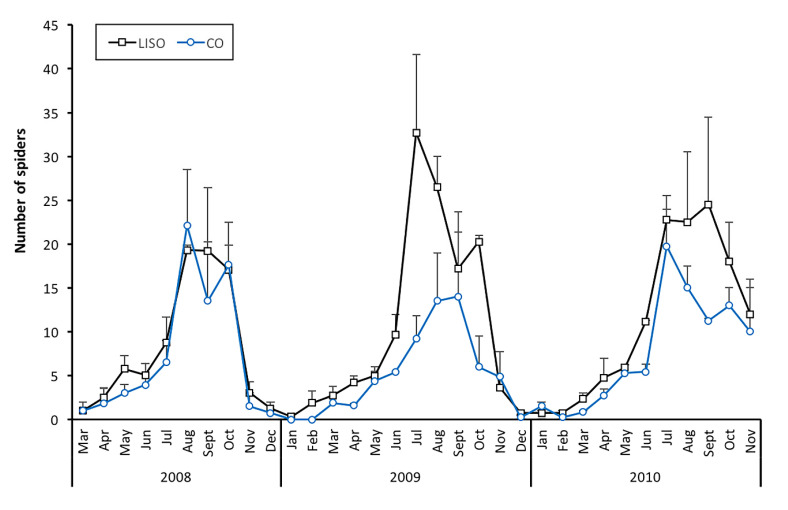
Monthly abundance (mean ± SE) of the number of spiders in the beating of 90 branches, collected throughout the period of study in orchards with two different intensity of spraying (LISO: low-intensity spraying orchards; CO: conventional orchards).

The trend in the abundance of spiders over time was similar between LISO and conventional orchards (Figure 2). In both types of orchards, the number of spiders on pear trees increased progressively in late spring or early summer (May–June), peaking in the warmest months of the year (from July to September). In 2008, similar density peaks of spiders were registered in August in LISO (19.3 ± 0.6 spiders in the beating 90 branches ± SE) and CO (22.1 ± 6.4). In contrast, higher peaks were registered in LISO than in conventional orchards in 2009 (LISO: 32.7 ± 8.9 in July; CO: 14.0 ± 9.7 in September) and 2010 (LISO: 24.5 ± 10.0 in September; CO: 19.7 ± 4.2 in July). In mid-autumn, the number of spiders on pear trees showed a generally sharp decline, leading to a number of captures close to zero at the end of the year and during the whole winter period (Figure 2).

A lower percentage of adults than immatures was always registered in the population of spiders throughout all the months of the year over the three years of the study, independently of the intensity of spraying. However, a higher percentage of adults was generally registered from April (20.5 ± 4.5%, mean ± SE of the three years) to June (25.5 ± 4.2%), with a sharp decline from July (7.1 ± 1.6%) to December (0%).

### 3.3. Abundance and Trend of Families/Guilds

The Philodromidae, Salticidae, and Cheiracanthiidae were the only three spider families that showed significantly different abundances in relation to the intensity of spraying (Table 2), being more abundant in LISO than in conventional orchards. Additionally, the abundance of the main families, with the exception of Philodromidae and Linyphiidae, differed significantly among years (Table 2). A high correlation was found between the prediction of GLMM and the experimental values for all the families of spiders (Supplementary Material, Table S3.1).

**Figure 3:**
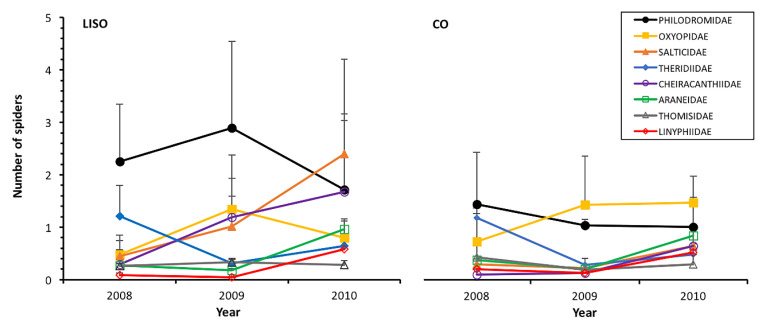
Annual abundance (mean ± SE, beating of 90 branches) of the different families of spiders in pear orchards over three years. LISO, low-intensity spraying orchards; CO, conventional orchards.

In the orchards with low-intensity of spraying, the philodromids were the most abundant family, reaching the highest density in 2009 (2.89 *±* 1.65 individuals in the beating 90 branches, annual mean ± SE) and lowest numbers in 2010 (Figure 3). In conventional orchards, the abundance of this family peaked in 2008 (1.42 ± 0.97) and decreased thereafter (Figure 3). Salticidae and Cheiracanthiidae showed increasing abundance from 2008 to 2010 in both types of orchards, peaking in 2010 in both LISO (Salticidae: 2.40 ± 1.80; Cheiracanthiidae: 1.68 ± 1.48) and CO (Salticidae: 0.64 ± 0.40; Cheiracanthiidae: 0.64 ± 0.32) (Figure 3). The Tukey’s test revealed that these two families were significantly more abundant in 2010 than in previous years (*p* < 0.001). Among the families that were not significantly affected by the intensity of spraying, the most abundant ones were Oxyopidae and Theridiidae. The abundance of both families differed significantly among years but their peaks were very similar between treatments (Oxyopidae: LISO: 1.34 ± 0.58; CO: 1.46 ± 0.50; Theridiidae: LISO: 1.21 ± 0.59; CO: 1.17 ± 0.07) (Figure 3). The remaining families showed low abundance throughout the study period in both types of orchards, with the linyphiids being the only one not significantly affected by the year of study (Table 2).

**Figure 4:**
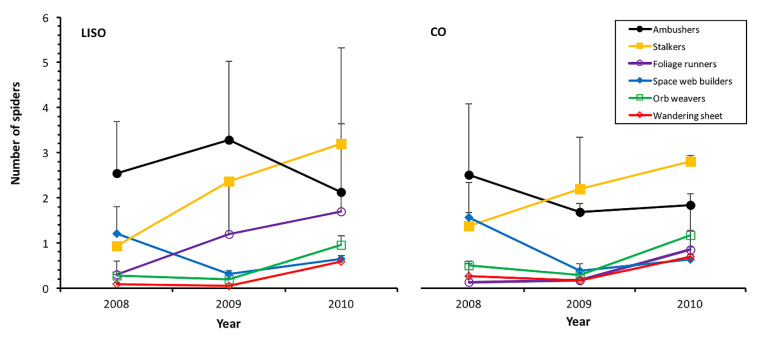
Annual abundance (mean ± SE, beating of 90 branches) of the different guilds of spiders in pear orchards over three years. LISO, low-intensity spraying orchards; CO, conventional orchards.

Ambushers and foliage runners were the only two guilds of spiders significantly influenced by the intensity of spraying, with higher abundances in low-intensity spraying orchards than in conventional orchards (Table 3, Figure 4). A high correlation was found between the prediction of GLMM and the experimental values for all the guilds of spiders (Supplementary Material, Table S3.2). The highest peak of ambushers occurred in 2009 in LISO (3.29 ± 1.74), while in CO, the peak was much lower (1.87 ± 1.18) and took place in 2008. Foliage runners, mostly represented by the family Cheiracanthiidae, showed increasing abundance in both types of orchards, peaking in 2010 in both cases (LISO: 1.70 ± 1.46; CO: 0.64 ± 0.32) (Figure 4). A significantly lower abundance of this guild was registered in 2008 than in 2009 and 2010 (Tukey’s test, *p* < 0.001). Among the guilds that showed similar abundance between treatments, stalkers constituted the most numerous one. As foliage runners, they showed increasing abundance over time in both types of orchards, peaking in 2010 (LISO: 3.20 ± 2.12; CO: 2.10 ± 0.10) and at a lower abundance in 2008 than in 2009 (Tukey’s test, *p* = 0.024) and 2010 (Tukey contrast, *p* = 0.004). Other relevant guilds showed some significant variation in their abundance among years but without significant differences in relation to the intensity of spraying (Table 3, Figure 4).

### 3.4. Structure of the Assemblage of Spiders

**Figure 5:**
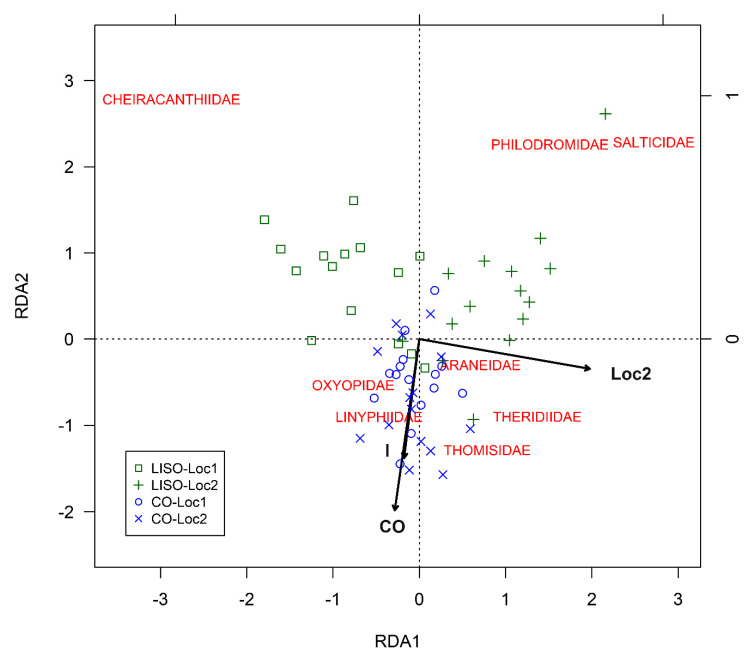
Redundancy analyses (RDA) for the monthly abundance of spider families found in pear orchards as a function of intensity of spraying, locality, and their interaction. RDA1, first constrained ordination axis; RDA2, second constrained axis; LISO, low-intensity spraying orchards; CO, conventional orchards; Loc2, locality 2; I, interaction.

In the RDA analysis for the families of spiders, most of the data points (i.e., RD1 and RD2 scores for the monthly average of the number of individuals in the different families of spiders, beating of 90 branches) from LISO grouped on the positive side of RDA2, while most of the data points from conventional orchards did so on the negative side of RDA2 (Figure 5). RDA1 separated the samples from locality 1 and locality 2 for LISO but not for conventional orchards. The RDA model was found out to be highly significant (Permutation test, F = 5.72, df = 3, 42, *p* < 0.001). The three terms included in the RDA model were found to be highly significant: (1) intensity of spraying (F = 4.66, df = 1, 42, *p* < 0.001), (2) locality (F = 6.24, df = 1, 42, *p* < 0.001), and (3) the interaction term (F = 6.26, df = 1, 42, *p* < 0.001). The three constrained axes were highly significant: RDA1 (F = 9.13, df = 1, 42, *p* < 0.001), RDA2 (F = 4.64, df = 1, 42, *p* < 0.001), and RDA3 (F = 3.40, df = 1, 42, *p* < 0.001). These three axes together explained 29.0% of the variance in the experimental data: RDA1 (15.4%), RDA2 (7.8%), and RDA3 (5.7%). A higher percentage of the variance (41.7%) was explained by the unconstrained axes: PC1 (16.3%), PC2 (15.5%), and PC3 (9.9%). The Cheiracanthiidae, Salticidae, and Philodromidae families were positively correlated with low-intensity of spraying, while Thomisidae, Linyphiidae, Oxyopidae, and Theridiidae seemed to be more associated with conventional orchards (Figure 5). In LISO, most of the data points from the two localities grouped on either the negative or the positive side of RDA1 and were correlated to different families of spiders (i.e., Cheiracanthiidae (Loc1) and Philodromidae/Salticidae (Loc2)). Data points from conventional orchards did not show any evident segregation (Figure 5).

## 4. Discussion

Spiders are among the most diverse and abundant predacious arthropods in most agroecosystems, including orchards [10,34]. This group constitutes a great part of the polyphagous predatory complex, being able to display, on some occasions, a significant controlling power on pest insects [14,29,45]. In the present study, it has been found that the canopy of Mediterranean pear orchards hosts a rich community of spiders belonging to 13 different families and 51 genera. This richness of genera is similar to that reported for other communities of spiders in fruit tree orchards [4,31,46]. Just five genera accounted for 72% of the total captures, which also agrees with the dominance of a few taxa reported for other communities of spiders in agroecosystems [47]. Philodromidae, mainly represented by the genus *Philodromus*, was the most numerous family. This genus has also been cited among the most abundant in the canopy of fruit orchards in other areas of Europe [34,46,48,49,50], including pear trees [3,31,51]. Philodromids have been reported to be active in pear orchards throughout the year, lowering the population growth of *C. pyri* in its initial phases [51,52,53]. The second most abundant family in our study was Oxyopidae, which is also very abundant in agricultural crops [54], including pome fruit orchards [4]. Oxyopidae are adapted to warm regions [13,55] and have been found to keep several pests under control in different agroecosystems [56,57]. Salticids, theridiids, and cheiracanthiids were also well represented in our samples, and to a lesser extent, other groups such as araneids, thomisids, and linyphiids. Salticids, especially the genus *Icius*, have also been reported to be abundant in other Mediterranean crops, such as olive and citrus trees [46,48]. In contrast, theridiids and lyniphiids seem to be more represented in orchards of cold-temperate areas [31,35]. These findings agree with the reduction of the proportions of theridiids and linyphiids in spider assemblages at low latitudes [3,58]. Cheiracanthiids, for their part, were only represented by the genus *Cheiracanthium*, which is a common genus in Spanish agroecosystems [46,59,60] and includes species that have been identified as effective hunters of some pest insects [61]. In summary, it can be said that the community of pear orchards in southern Spain is dominated by wandering spiders (i.e., philodromids, oxyopids, salticids, and cheiracanthiids), like it has been reported for other fruit orchards in the Iberian Peninsula [46,48,58]. This is in contrast with the composition of Arachnofauna in pear orchards in regions with a cold-temperate climate, which is usually dominated by web builders, like theridiids, araneids, and linyphiids [3,27,31,35].

The population dynamics of spiders on the canopy of pear trees showed a distinct annual pattern, with density peaks during the summer months and relatively low abundances during the rest of the year. This agrees with population dynamics reported by other authors [40,62,63,64]. The population of spiders was characterized by a high proportion of juveniles, which seems to be a constant in natural and managed ecosystems [4,46,63,65,66,67]. However, the proportion of juveniles was lower in spring than in summer and autumn, which is consistent with most of the studies in pome fruit orchards [4,34,35,40,64]. The increase in juveniles in summer may be originated from the reproduction of both the individuals that overwinter in orchards and those migrating from the surroundings habitats [64]. The population dynamics of spiders is known to be affected by the frequency and timing of the application of pesticides [30,34,35,36,58,68,69]. In southern Spain, the spring population of reproductive adults may suffer greatly from the intensification of spraying against pear psyllids during this period, which may explain, at least in part, the lower density peaks registered in summer in conventional orchards in the present study.

The application of pesticides, particularly of broad-spectrum formulations, has been largely reported to reduce the abundance and diversity of spiders in fruit tree orchards [8,35,36,37]. These compounds have been found to alter the reproduction, development, and foraging behavior of spiders [70,71,72], not only causing direct mortality but also diminishing their efficiency as biological control agents [34,54,73]. Moreover, pesticides may also reduce spider populations indirectly, for instance, by depleting their prey [34]. In the present research, the lowest richness of genera and abundance of spiders were generally registered in pear orchards with the highest intensity of spraying, with a variable impact depending on the families of spiders. Philodromidae, Salticidae, and Cheiracanthiidae were the families that experienced a significant population reduction in the orchards with a higher intensity of spraying. Earlier authors reported a negative effect of pesticides on philodromids [74]; although, others have reported a certain resistance to pesticides in some *Philodromus* species [31,75,76]. Salticids have been reported as extremely susceptible to pesticides in orchards in warm regions worldwide [46,58,77]. The impact of pesticides on cheiracanthiids and particularly on *Cheiracanthium* species is little known, but some populations have been found to exhibit a certain resistance to insecticides [78]. However, our results and other previous studies [4,46] suggest that this genus could be highly sensitive to chemicals. Other families such as Araneidae, Linyphiidae, and Thomisidae were not significantly affected by the intensity of spraying. In the study area, paraffin oil and abamectin are the most frequently used products for pest control. Bajwa and Aliniazee [79] found that summer oil had little effect on spiders. However, although mineral oils have a short-term residual activity, most predators have been found to be killed on contact or by direct spraying [79,80]. We found no information on the effect of abamectin on spiders. However, this compound has been proved to be highly toxic for several species of natural enemies (https://www.biobestgroup.com/en/side-effect-manual).

The impact of pesticides on spiders has been reported to be related to their foraging strategies, and it is known to affect some guilds more severely than others [8,35,37,81]. Wandering spiders with a diurnal foraging pattern have been generally reported to be more negatively affected by pesticides than web builders; possibly, because they might be more exposed to the physical impact of spraying and the toxicity of chemicals, which could be minimized by nocturnal habits or the protection of webs [32,33,58]. In agreement with these statements, in the present research, some diurnal wandering spiders, such as ambushers (i.e., philodromids) and stalkers (i.e., salticids), were significantly affected by the intensity of spraying, while no significant effect was found on web builders (i.e., theridiids, areaneids, and linyphiids). Philodromids usually adopt a cryptic defense behavior that is of little use against the physical or toxic effects of pesticides [82], while salticids, being strictly diurnal [83], have a higher chance of being impacted by spraying than crepuscular or nocturnal species. In relation to the guilds less susceptible to pesticides, Pekár [74] observed that some araneids survived against broad-spectrum insecticides by hiding under leaves when they are out of their webs. However, they are considered susceptible to insecticides because they recycle their webs through ingestion [32]. The lower effects of pesticides on some web builders, such as theridiids, have been attributed to a protective effect of their complex tent-like webs, which include a retreat inside [32,33,58]. In contrast to these observations, Khan [37] found that web-building spiders of the families Theridiidae and Tetragnathidae were severely affected by the application of pesticides in apple orchards of Kashmir. Contrary to the expectations in relation to their foraging behavior, some families of ambushers (i.e., thomisids) and stalkers (i.e., oxyopids) showed similar abundance in biological and conventional orchards. In addition, some foliage runners with nocturnal habits (i.e., cheiracanthiids) were significantly less abundant in the more intensively sprayed orchards. The low impact of spraying on thomisids and oxyopids could be explained by the ability of these spiders to commute between the canopy of trees and understory vegetation, avoiding direct exposure to chemicals [4]. In contrast, the findings of the present study suggest that the nocturnal habits of cheiracanthiids do not protect them against the action of pesticides and it is quite likely that being foliage runners increases their exposure to chemicals.

Other factors apart from the intensity of spraying may have accounted for the differences in abundance and structure of the spider assemblages registered in pear orchards in the present research. RDA analyses showed that a good deal of the variance in the experimental data was explained by the intensity of spraying and locality, but a higher percentage was attributed to unaccounted environmental factors. No significant differences in the abundance of spiders were registered between orchards with low-intensity of spraying and conventional orchards in years with great differences in their spraying calendar (i.e., 2008), while differences were registered between years when the two types of orchards differed less in the intensity of spraying (i.e., 2009). This could be explained by the history of treatment of orchards and/or by a delayed effect of spraying, due to the effect that the lowering in the number of overwintering breeders in the previous year may have on the population growth of the following annual cycle. The composition and species dominance in spider assemblages could also be determined by regional differences in environmental variables, such as climate, soil, and the surrounding vegetation [40,84,85,86]. In this regard, Bogya et al. [3] stated that pesticide application can significantly influence the populations of spiders in orchards but this factor is of moderate importance compared with the effect of geographical location. In this study, the dominance of cheiracanthiids in one of the orchards with low-intensity of spraying and of philodromids/salticids in the other could be due to local environmental conditions.

## 5. Conclusions

The present research constitutes, to our knowledge, the first study on the structure, richness, abundance, and population dynamics of spider assemblages in the canopy of Mediterranean pear orchards. These assemblages were characterized by a high number of species, but just a few genera accounted for most of the abundance of spiders. Our results confirm that the intensity of spraying has a negative impact both on the abundance and richness of genera. However, the impact of spraying varied depending on the family of spiders, with Philodromidae, Salticidae, and Cheiracanthiidae significantly affected. *Philodromus* spp. has been reported to use psyllids as a major source of prey during winter, and as one of the key predators to reduce the psyllid populations during this period in pear orchards [51,52,53]. Therefore, the reduction of the overwintering population of philodromids could translate into higher psyllid outbreaks in spring. The different impact of pesticides on spiders could be explained, to a certain degree, by their foraging mode. According to the expectations, some wandering spiders, such as philodromids, salticids, and cheiracanthiids, were negatively affected, while web builders, such as theridiids, areaneids, and linyphiids, were not. Contrary to expectations, some families of wandering spiders, such as thomisids and oxyopids, showed similar abundance in low-intensity spraying and conventional orchards. As a result of the different degree of susceptibility of spider families to pesticides, the structure of the communities of spiders is expected to vary in function of the pest management strategy. The variation among years and between localities suggests that other environmental factors may have a great effect on populations and the structure of spider assemblages in Mediterranean pear orchards. Further studies on the role of spiders on pest control, the factors that affect their abundance and diversity, and the interaction among species are strongly recommended to understand the dynamics of the community of arthropods in pear orchards and to enhance their management as biological pest control agents.

## Figures and Tables

**Table 1:** Number of individuals collected in different families of spiders grouped in guilds, both basic and according to the criteria of Uetz et al. [44], in low-intensity spraying (LISO) and conventional (CO) pear orchards.

Basic Guild	Guild	Family	LISO	CO
WEB BUILDERS	Space web builders	THERIDIIDAE	116	98
	Orb weavers	ARANEIDAE	80	81
		ULOBORIDAE	1	3
	Wandering sheet/Tangle weavers	LINYPHIIDAE	39	50
WANDERING SPIDERS	Ambushers	PHILODROMIDAE	448	204
		PISAURIDAE	15	14
		THOMISIDAE	55	55
	Stalkers	SALTICIDAE	243	67
		OXYOPIDAE	175	230
	Foliage runners	CLUBIONIDAE	1	0
		CHEIRACANTHIIDAE	197	49
		SPARASIDAE	1	0
	Ground runners	GNAPHOSIDAE	9	3

**Table 2:** Statistics of generalized linear mixed models (GLMM) for the effect of intensity of spraying, year, and their interaction on the abundance of the main spider families. χ^2^ = Chi square values (degrees of freedom within brackets).

	Intensity of Spraying		Year		Interaction	
Family	χ^2^(1)	*p*-Value	χ^2^(2)	*p*-Value	χ^2^(2)	*p*-Value
Philodromidae	9.253	0.002	2.158	0.340	1.023	0.600
Oxyopidae	1.992	0.158	13.656	0.001	1.682	0.431
Salticidae	20.570	<0.001	15.422	<0.001	1.493	0.474
Cheiracanthiidae	21.872	<0.001	16.956	<0.001	2.959	0.228
Theridiidae	0.930	0.335	39.648	<0.001	0.630	0.730
Araneidae	0.373	0.541	7.803	0.020	2.045	0.360
Thomisidae	2.248	0.134	7.783	0.020	3.254	0.196
Linyphiidae	3.150	0.076	3.918	0.141	2.002	0.367

**Table 3:** Statistics of generalized linear mixed models (GLMM) for the effect of intensity of spraying, year, and their interaction on the abundance of the main spider guilds. χ^2^ = Chi square values (degrees of freedom within brackets).

	Intensity of Spraying		Year		Interaction	
Guild	χ^2^(1)	*p*-Value	χ^2^(2)	*p*-Value	χ^2^(2)	*p*-Value
Ambushers	6.991	0.008	2.951	0.229	2.588	0.274
Stalkers	2.232	0.135	12.258	0.002	1.160	0.560
Foliage Runners	35.732	<0.001	29.338	<0.001	5.8179	0.054
Space web builders	0.982	0.322	53.939	<0.001	0.247	0.884
Orb Weavers	0.185	0.667	15.496	<0.001	1.533	0.465
Tangle Weavers	0.148	0.701	6.038	0.049	3.329	0.189

## Supplementary Materials

The following are available online at https://www.mdpi.com/2075-4450/11/9/553/s1, Table S1: Pesticide spray calendar in the studied pear orchards. Table S2: Number of spiders of the different genera collected by the beating of pear tree branches in low-intensity spraying orchards and conventional orchards. Tables S3.1 and S3.2. Validation of GLMM for the abundance of the families and guilds of spiders in pear orchards.
