# Supplementary material for: Structure of the Assemblages of Spiders in Mediterranean Pear Orchards and the Effect of Intensity of Spraying
Source: Insects. 2020 Aug 20;11(9):553. doi: 10.3390/insects11090553 (PMC7564418; doi:10.3390/insects11090553)
Supplement: Supplementary file 1 [file insects-11-00553-s001.zip › TableS1_R1.docx]

**Table S1.** Pesticide spray calendar in the studied pear orchards. *LISO: Low-intensity spraying orchard; CO: Conventional orchard; Loc: Locality.

| **Orchard*** | **Year** | **Date** | **Active ingredient** |
| --- | --- | --- | --- |
| LISO_Loc1 | 2008 | - | - |
|  | 2009 | March 10 | Paraffinic oil 83% |
|  |  | May 19 | Paraffinic oil 83% |
|  | 2010 | February 2 | Paraffinic oil 83% |
| CO_Loc1 | 2008 | January 8 | Calcium polysulphur 18.5% |
|  |  | February 27 | Paraffinic oil 83% |
|  |  | March 1 | Paraffinic oil 83% + Kaolin 95% |
|  |  | April 14 | Paraffinic oil 83% + Kaolin 95% |
|  |  | May 2 | Paraffinic oil 83% |
|  |  | May 5 | Paraffinic oil 83% + *Beauveria bassiana* |
|  |  | May 10 | Paraffinic oil 83% + *Beauveria bassiana* |
|  |  | August 29 | Paraffinic oil 83% + *Beauveria bassiana* |
|  |  | November 4 | Paraffinic oil 83% |
|  | 2009 | February 12 | Calcium polysulphur 18.5% |
|  |  | March 2 | Paraffinic oil 83% |
|  |  | March 10 | Paraffinic oil 83% |
|  |  | November 11 | Paraffinic oil 83% + Cuprocalcium sulfate 20% |
|  | 2010 | February 2 | Paraffinic oil 83% |
|  |  | March 11 | Paraffinic oil 83% + Copper oxide 50% |
| LISO_Loc2 | 2008 | - | - |
|  | 2009 | - | - |
|  | 2010 | - | - |
| CO_Loc2 | 2008 | March 29 | Fenoxycarb 25% |
|  |  | April 14 | Acrinathrin 7.5%+ Fenoxycarb 25% |
|  |  | May 24 | Abamectin 1.8% + Paraffinic oil 83% |
|  |  | June 6 | Abamectin 1.8% + Paraffinic oil 83% |
|  |  | October 3 | Abamectin 1.8% + Paraffinic oil 83% |
|  |  | December 23 | Paraffinic oil 83% |
|  | 2009 | March 10 | Paraffinic oil 83% + Copper + Kaolin 95% |
|  |  | April 3 | Abamectin 1,8%+Fenoxycarb 25%+ Paraffinic oil 83% |
|  |  | April 4 | Abamectin 1,8%+Fenoxycarb 25%+ Paraffinic oil 83% |
|  |  | May 25 | Abamectin 1.8% + Paraffinic oil 83% |
|  |  | June 3 | Abamectin 1.8% + Paraffinic oil 83% |
|  |  | October 6 | Abamectin 1.8% + Paraffinic oil 83% |
|  | 2010 | March 23 | Paraffinic oil 83% |
|  |  | March 31 | Abamectin 1.8% |
|  |  | April 29 | Paraffinic oil 83% |
|  |  | May 20 | Paraffinic oil 83% |
