# Supplementary material for: Structure of the Assemblages of Spiders in Mediterranean Pear Orchards and the Effect of Intensity of Spraying
Source: Insects. 2020 Aug 20;11(9):553. doi: 10.3390/insects11090553 (PMC7564418; doi:10.3390/insects11090553)
Supplement: Supplementary file 1 [file insects-11-00553-s001.zip › Table_S2_R1.docx]

**Table S2.** Number of spider of the different genera collected by the beating of pear tree branches in low-intensity spraying orchards (LISO) and conventional orchards (CO). (Loc. 1: Locality 1; Loc. 2: Locality 2). N, total number of individuals collected.

| **Taxon** | | **Orchard** | | | | **N** |
| --- | --- | --- | --- | --- | --- | --- |
| **Family** | **Genus** | **LISO**  **Loc. 1** | **LISO**  **Loc. 2** | **CO**  **Loc. 1** | **CO**  **Loc. 2** |  |
| **Araneidae** | *Araneus* Clerck, 1757 | 1 | 1 | 1 | 0 | 3 |
|  | *Araniella* Chamberlin & Ivie, 1942 | 0 | 0 | 2 | 0 | 2 |
|  | *Cyclosa* Menge, 1866 | 1 | 0 | 0 | 0 | 1 |
|  | *Mangora* O.P.-Cambridge, 1889 | 2 | 4 | 3 | 3 | 12 |
|  | *Neoscona* Simon, 1864 | 26 | 45 | 49 | 23 | 143 |
| **Cheiracanthiidae** | *Cheiracanthium* C.L. Koch, 1839 | 191 | 6 | 37 | 12 | 246 |
| **Clubionidae** | *Clubiona* Latreille, 1804 | 1 | 0 | 0 | 0 | 1 |
| **Gnaphosidae** | *Aphantaulax* Simon, 1878 | 1 | 2 | 0 | 2 | 5 |
|  | *Gnaphosa* Latreille, 1804 | 0 | 2 | 0 | 0 | 2 |
|  | *Leptodrassus* Simon, 1878 | 0 | 1 | 0 | 0 | 1 |
|  | *Zelotes* Gistel, 1848 | 1 | 2 | 0 | 1 | 4 |
| **Linyphiidae** | *Agyneta* Hull, 1911 | 1 | 1 | 1 | 2 | 5 |
|  | *Frontinellina* van Helsdingen, 1969 | 1 | 0 | 2 | 0 | 3 |
|  | *Lepthyphantes* Menge, 1866 | 0 | 0 | 1 | 0 | 1 |
|  | *Prinerigone* Millidge, 1988 | 0 | 0 | 1 | 0 | 1 |
|  | *Tenuiphantes* Saaristo & Tanasevitch, 1996 | 3 | 1 | 0 | 8 | 12 |
|  | *Walckenaeria* Blackwall, 1833 | 1 | 0 | 0 | 0 | 1 |
|  | Linyphiidae_Gen01 | 11 | 11 | 14 | 11 | 47 |
|  | Linyphiidae_Gen02 | 0 | 0 | 1 | 1 | 2 |
|  | Linyphiidae_Gen03 | 1 | 0 | 2 | 0 | 3 |
|  | Linyphiidae_Gen04 | 1 | 0 | 1 | 2 | 4 |
|  | Linyphiidae_Gen05 | 4 | 2 | 2 | 0 | 8 |
|  | Linyphiidae_Gen06 | 1 | 0 | 1 | 0 | 2 |
| **Oxyopidae** | *Oxyopes* Latreille, 1804 | 110 | 65 | 149 | 81 | 405 |
| **Philodromidae** | *Philodromus* Walckenaer, 1826 | 125 | 306 | 114 | 77 | 622 |
|  | *Pulchellodromus* Wunderlich, 2012 | 2 | 3 | 0 | 2 | 7 |
|  | *Thanatus* C.L. Koch, 1837 | 2 | 8 | 8 | 3 | 21 |
|  | *Tibellus* Simon, 1875 | 0 | 2 | 0 | 0 | 2 |
| **Pisauridae** | *Pisaura* Simon, 1886 | 3 | 12 | 4 | 10 | 29 |
| **Salticidae** | *Chalcoscirtus* Bertkau, 1880 | 0 | 2 | 0 | 0 | 2 |
|  | *Evarcha* Simon, 1902 | 2 | 35 | 0 | 4 | 41 |
|  | *Heliophanus* C.L. Koch, 1833 | 0 | 0 | 1 | 0 | 1 |
|  | *Icius* Simon, 1876 | 23 | 131 | 28 | 8 | 190 |
|  | *Menemerus* Simon, 1868 | 0 | 1 | 0 | 0 | 1 |
|  | *Phlegra* Simon, 1876 | 0 | 2 | 0 | 0 | 2 |
|  | *Salticus* Latreille, 1804 | 8 | 1 | 9 | 1 | 19 |
|  | *Talavera* Peckham & Peckham, 1909 | 0 | 1 | 0 | 1 | 2 |
|  | *Thyene* Simon, 1885 | 3 | 34 | 2 | 13 | 52 |
| **Sparasidae** | *Olios* Walckenaer, 1837 | 0 | 1 | 0 | 0 | 1 |
| **Theridiidae** | *Enoplognatha* Pavesi, 1880 | 6 | 3 | 8 | 2 | 19 |
|  | *Euryopis* Menge, 1868 | 1 | 18 | 0 | 9 | 28 |
|  | *Kochiura* Archer, 1950 | 1 | 0 | 0 | 0 | 1 |
|  | *Platnickina* Kocak & Kemal, 2008 | 8 | 7 | 9 | 15 | 39 |
|  | *Simitidion* Wunderlich, 1992 | 3 | 0 | 0 | 0 | 3 |
|  | *Steatoda* Sundevall, 1833 | 12 | 35 | 13 | 25 | 85 |
|  | *Theridion* Walckenaer, 1805 | 8 | 14 | 8 | 9 | 39 |
| **Thomisidae** | *Runcinia* Simon, 1875 | 5 | 8 | 2 | 6 | 21 |
|  | *Synema* Simon, 1864 | 10 | 11 | 12 | 7 | 40 |
|  | *Thomisus* Walckenaer, 1805 | 0 | 6 | 0 | 0 | 6 |
|  | *Xysticus* C.L. Koch, 1835 | 7 | 8 | 10 | 18 | 43 |
| **Uloboridae** | *Uloborus* Latreille, 1806 | 1 | 0 | 0 | 3 | 4 |
| Total |  | 588 | 792 | 495 | 359 | **2234** |
