# Supplementary material for: Structure of the Assemblages of Spiders in Mediterranean Pear Orchards and the Effect of Intensity of Spraying
Source: Insects. 2020 Aug 20;11(9):553. doi: 10.3390/insects11090553 (PMC7564418; doi:10.3390/insects11090553)
Supplement: Supplementary file 1 [file insects-11-00553-s001.zip › Table_S3_Validation_GLMM_models.docx]

**TABLE S3**

**TABLE S3.1** Validation of GLMM for the abundance of the families of spiders in pear orchards. Coeff.= Pearson's coefficient of correlation; df= degree of freedom.

| **Family** | **Coeff.** | **t-value** | **df** | **P-value** |
| --- | --- | --- | --- | --- |
| Philodromidae | 0.516 | 4.59 | 58 | <0.001 |
| Oxyopidae | 0.795 | 9.97 | 58 | <0.001 |
| Salticidae | 0.844 | 11.98 | 58 | <0.001 |
| Cheiracanthiidae | 0.915 | 17.25 | 58 | <0.001 |
| Theridiidae | 0.733 | 8.20 | 58 | <0.001 |
| Araneidae | 0.377 | 3.10 | 58 | 0.003 |
| Thomisidae | 0.501 | 4.41 | 58 | <0.001 |
| Linyphiidae | 0.565 | 5.21 | 58 | <0.001 |

**TABLE S3.2.** Validation of GLMM for the abundance of the guilds of spiders in pear orchards. Coeff.= Pearson's coefficient of correlation; df= degree of freedom.

| **Family** | **Coeff.** | **t-value** | **df** | **P-value** |
| --- | --- | --- | --- | --- |
| Ambushers | 0.548 | 4.99 | 58 | <0.001 |
| Stalkers | 0.628 | 6.139 | 58 | <0.001 |
| Foliage Runners | 0.936 | 20.28 | 58 | <0.001 |
| Space web builders | 0.867 | 13.25 | 58 | <0.001 |
| Orb Weavers | 0.469 | 4.05 | 58 | <0.001 |
| Tangle Weavers | 0.607 | 5.817 | 58 | <0.001 |
